# Surgical Management of Renal Cell Carcinoma: Comparisons of open and laparoscopic approaches

**DOI:** 10.18295/squmj.6.2024.035

**Published:** 2024-08-29

**Authors:** Noor N. Junejo, Najib AbuDraz, Shahid Aquil, Joseph K. Mathew, Ghalib Al Badaai, Mohammed S. Al-Marhoon, Khurram M. Siddiqui

**Affiliations:** Department of Surgery, Sultan Qaboos University Hospital, University Medical City, Muscat, Oman

**Keywords:** Nephrectomy, Heminephrectomy, Laparoscopy, Renal Cell Carcinoma, Oman

## Abstract

**Objectives:**

Renal cell carcinoma (RCC) is a leading urological malignancy with an age-standardised incidence rate of 2.5 per 100,000 per year in Oman. Experts are inclined towards the early detection and use of minimally invasive technology for the treatment of RCC. This study aimed report the shifting trend in the clinical presentation and management of RCC in Oman, comparing the outcomes of laparoscopic and open nephrectomy.

**Methods:**

This retrospective study included adult RCC patients from Sultan Qaboos University Hospital, Muscat, Oman, diagnosed from 2011–2022. Patient biodata, mode of presentation, diagnostic modality, final histopathology and details of treatment received including the perioperative outcomes were analysed.

**Results:**

A total of 56 patients that underwent surgical treatment for RCC, 34 underwent laparoscopic nephrectomy (LN) and 22 underwent open nephrectomy (ON). The mean ages in the LN and ON groups were 53.82 ± 13.44 years and 56.22 ± 15.00 years (*P* = 0.53), respectively. There were 47 patients of Omani descent and 9 patients were expatiates. The patients’ mean tumour size was 6.25 ± 3.16 cm and 9.23 ± 5.20 cm for the LN and ON groups, respectively; 55.35% of the RCC cases were incidentally diagnosed. A trend towards LN was observed.

**Conclusion:**

This study found a trend towards early diagnosis of RCC in Oman, with the majority of cancers being discovered incidentally in the studied period. LN is more commonly used in the surgical management of RCC with acceptable morbidity. These trends remain aligned with those found in the global literature on RCC.

**Advances in Knowledge**

*- Laparoscopic approach is considered the gold standard of care in the surgical management of kidney cancer*.
*- To the best of the authors’ knowledge, this study is the first large case series of laparoscopic and open nephrectomy from Oman. The increasing trend of incidentally discovered tumours is not only observed in Oman but also in other developed countries*.
*- This study found that laparoscopic nephrectomy is a safe and efficient oncological modality with respect to the treatment of renal tumours*.
*- This study will help in establishing a new standard of cancer-related care and also provide local reference for further studies*.

**Application to Patient Care**

*- This article explores the safety and efficiency of a minimally invasive approach in managing renal cancer for both large and small renal masses*.
*- The report confirms that both radical and partial nephrectomy can be safety performed in Oman’s local setup; thus, patients should be offered laparoscopic treatment as the first preference, unless there is a specific contraindication. This article also highlights the concomitant importance of abdominal imaging and incidental diagnosis of renal mass*.
*- Renal tumours should not be overlooked; most solid renal masses identified via imaging turn are malignant and their timely treatment facilitates an excellent cure rate*.

Renal Cell carcinoma (RCC) is the sixth most common tumour in males and tenth most common in females.^1^ In Oman, the age standardised incidence rate for kidney cancer is 4.0 in males and 1.9 in females. In fact, over the last 3 decades, there has been a rise in RCC diagnosis among people living in Oman.^2^ The international literature has attributed this rise to the increasing use of diagnostic imaging, owing to which most cancers are diagnosed incidentally.^3^ In 1991, laparoscopic nephrectomy was used to treat RCC for the first time; since then, advancements in minimally invasive surgical techniques have resulted in the laparoscopic approach being considered the best option for the surgical management of RCC.^3^^,^^4^ Subsequently, early diagnosis and effective surgical management of RCC has resulted in improved survival rates among those diagnosed with this disease.^5^ For small renal masses, partial nephrectomy (PN) is currently the standard treatment; however, owing to several anatomical and logistic factors, a large proportion of RCC cases remain unamenable to PN.^6^^,^^7^ In this context, the present study aimed analyse Oman’s RCC patients’ demographics, mode of clinical presentation as well as surgical and immediate oncological outcomes as well as compare the safety and efficiency of both laparoscopic and open nephrectomy regarding RCC treatment.

## Methods

This retrospective study was conducted at Sultan Qaboos University Hospital (SQUH), Muscat, Oman. The hospital’s information system was used to identify all patients reporting a diagnosis of RCC between 2011 and 2022. Patients under the age of 12 and those with other kidney cancers such as upper tract transitional cell carcinoma were excluded from the study sample. The variables that were recorded included patient biodata, mode of presentation, diagnostic modality, final histopathology and details of treatment received including the perioperative outcomes.

The intraoperative and postoperative complications were graded based on the Clavien-Dindo system.^8^ A Chi-square analysis of both the continuous and categorical variables were conducted. *P* <0.05 was considered statistically significant.

The study variables, both continuous and categorical, were subjected to a Chi-square analysis. A *P*-value < 0.05 was set as a level of significance.

This study received ethical approval from Sultan Qaboos University (MREC#2750).

## Results

A total of 56 patients were included in this study. Among them, 34 patients underwent laparoscopic nephrectomy (LN), 25 underwent laparoscopic radical nephrectomy and 9 patients underwent laparoscopic partial nephrectomy (LPN). Furthermore, 22 patients underwent open nephrectomy, 16 patients underwent open radical nephrectomy and 6 patients had open partial nephrectomy (OPN). In total, 47 patients were Omani and 9 patients were expatriates. There was no difference in the mean age of the patients. The mean tumour sizes of the selected patients were 6.25 cm ± 3.16 and 9.23 cm ± 5.20 for the laparoscopic and open nephrectomy (ON) groups, respectively. At the time of clinical presentation, 55.35% of the RCC cases were deemed incidentally diagnosed [Table 1].

In total, 15 patients underwent partial nephrectomy (9 = LPN, 6 = OPN) for polar tumours (the tumour was located at the lower pole in 8 patients and at the upper pole in 7 patients). It was observed that the mean estimated blood loss (EBL) during surgery was lower in the LN compared to the ON group (351.76 ± 400.98 mL versus 512.95 ± 616.20 mL). Moreover, the mean surgery time was found to be slightly shorter in the LN than in the ON group (205 ± 73.37 min versus 217.82 ± 161.87 min). However, none of these differences were considered statistically significant [Table 2].

The complications were graded using the Clavien-Dindo system. This study found that 4 patients undergoing LN and 5 patients undergoing ON had grade 1–2 complications. Meanwhile, grade 3–4 complications were observed in 3 LN and 4 ON patients (*P* = 0.23). The difference in the mean postoperative duration of hospital stay was statistically significant when comparing the LN to ON group (5 days versus 7 days; *P* = 0.04). The clear cell type of RCC was found in as many as 34 cases (60.71%), while non-clear cell RCC was found in 22 cases. Among the patient with non-clear cell RCC, 12 were considered cases of chromophobe carcinoma, 7 were considered cases of papillary carcinoma and 1 was considered a case of oncocytoma. Notably, 2 unique cases of primary squamous cell carcinoma were also found. The TNM stage of the tumor is detailed in Table 3. It shows that the majority of patients had organ-confined disease (T1-T2). There were 6 cases of RCC with tumor thrombus extending to the inferior vena cava (T3b-T3c). Additionally, 2 patients with metastatic disease underwent cytoreductive surgery.

**Figure 1:**
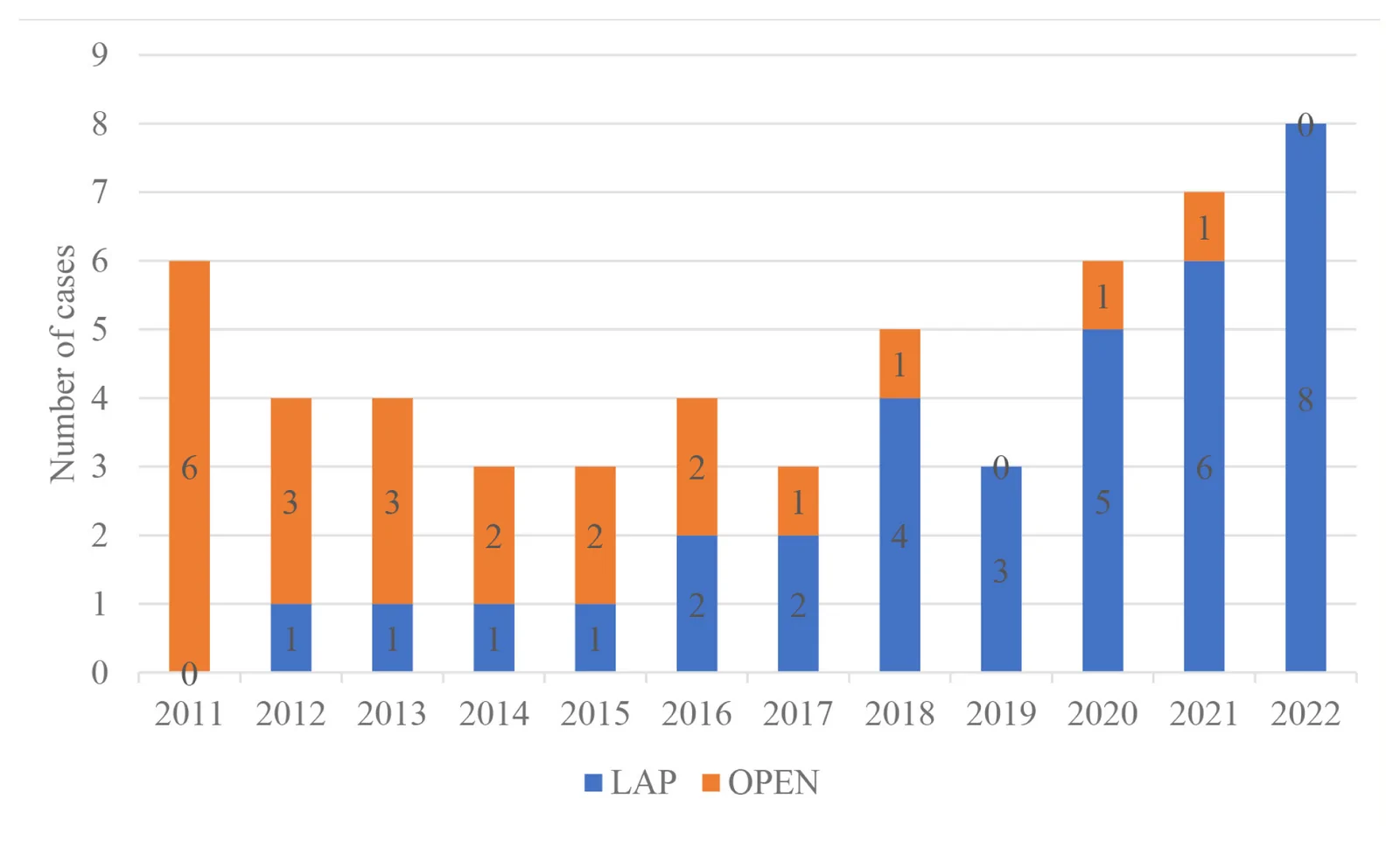
Management of renal cell carcinoma (laparoscopic or open approach) at Sultan Qaboos University Hospital, Muscat, Oman. *LAP = laparoscopic*.

The Fuhrman nuclear grade results were available for 53 patients and showed that most patients had grade 2 (49.05%), followed by grade 3 (43.39%), grade 1 (7.54%) and grade 4 (3.77%). There has been an increasing preference for LN at SQUH in recent years [Figure 1]. The median follow-up was 40 months post-surgery and the overall survival rate was 95%.

## Discussion

In the West, the diagnosis and treatment of RCC have significantly changed in recent years due to the widespread use of imaging modalities and dispersion of minimally invasive technology.^9^ Therefore, this study aimed to analyse the patterns of presentation and the changing trends of treatment regarding RCC in Oman. In 2019, the Oman cancer registry reported on the rising kidney cancer incidence in the nation, with such cases almost doubling between 2009 and 2019 (from 26 to 59, respectively).^2^ This increase in incidence without a corresponding increase in mortality is arguably a reflection of more cases being, not only, diagnosed incidentally but also at an earlier stage, a trend that is well established in other parts of the world.^10^ This study found that 55.35% of the included patients were incidentally diagnosed. In line with the international literature on RCC, the present study also reported early-stage cancer in the majority of its selected patients with 51.78% and 16.07% cases being diagnosed with cancer at clinical stages T1 and T2.^11^ Moreover, there were 2 rare cases of primary squamous cell carcinoma.^12^

Most of the cancer cases observed in this study reported an early stage of cancer (<T3a). A total of 6 patients had renal vein/IVC thrombus and required open IVC exploration. Moreover, there were 2 patients who had metastatic disease and underwent cytoreductive nephrectomy. Subsequently, this study found that no patient who underwent radical or partial nephrectomy had a positive surgical margin and required revision surgery.

The first laparoscopic nephrectomy was performed in 1991 and has become part of a surgical trend that has now dispersed around the world; this minimally invasive technique is the gold standard regarding the surgical treatment of RCC.^13^ The present study reported that LN was being performed more frequently than ON which indicates a trend leaning towards LN. The temporal delay in the adoption of the laparoscopic approach (as compared to its adoption in the Western world) was attributed to the concomitant training of human resources and logistics. A recent trend at the studied institute showed that open radical nephrectomy is only performed for cases with very large tumours, large renal vein thrombus or inferior vena cava thrombus. In this study’s sample, there were 6 patients who presented with T3b-c disease. On the other hand, for smaller-tumour RCC cases, the current standard of care mandates partial nephrectomy, which reportedly ensures excellent oncologic control without the morbidity of functional nephrons.^13^ Hence, the authors of this study performed partial nephrectomies in 15 cases, including 9 laparoscopic nephrectomies and 6 open nephrectomies.

Currently, nephron-sparing surgery is preferred with regard to a laparoscopic/robotic approach to RCC treatment.^14^ However, in certain anatomical locations, patient comorbidity and availability of robotic assistance are major hurdles to the laparoscopic approach.^15^ Currently, experts in Oman do not have access to the robotic system; thus, they consider open partial nephrectomy as a safer alternative for RCC cases.^16^

To establish the safety and efficacy of the LN approach, the present study observed the operative variables such as duration of surgery, EBL, hospital stay and surgical complications as well as the differences between the LN and ON approaches. The mean duration of surgery was slightly shorter in the LN group (205 min versus 217.82 min) and EBL was also less in the LN group (351.76 mL versus 512.95 mL) compared to the ON group; however, these differences were not statistically significant. As expected, the ON approach entailed a longer mean duration of hospital stay than the LN approach (5 versus 7 days; *P* = 004). In addition, the mean tumour size was found to be statistically different among the LN and ON groups (6.25 cm versus 9.23 cm; *P* = 0.02). These findings aligned with Tannus *et al*.’s work.^17^

The surgical complications that arose were graded according to the Clavien-Dindo system; this study found a total of 3 patients in the LN group developed complications, whereas 5 patients who underwent treatment using the ON approach developed complications; however, this difference was not statistically significant (*P* = 0.230). There were 2 cases that required a change in approach (from a pure laparoscopic approach to a hand-assisted laparoscopic approach). The results of this study align with the recent findings of a large cohort study which also showed a lower complication rate for a minimally invasive approach to RCC treatment.^18^

The median follow-up in this study was 40 months post-surgery and the overall survival rate at 40 months post-surgery was observed to be 95%. Junejo *et al*.’s study, conducted in Saudi Arabia, reported a similar survival rate.^19^

## Conclusions

This study found that a greater number of patients were diagnosed with RCC incidentally and with small renal masses, which is in concordance with the available literature on RCC cases. The surgical treatment of RCC at the study’s institution revealed a changing trend: an increasing number of patients were being treated for RCC using the laparoscopic approach (for both radical and partial nephrectomy). The concomitant intraoperative data and the complication rates also supported the safety and efficacy of the laparoscopic approach at this institute. Therefore, the widespread use of imaging results in the diagnosis of early stage RCC and a minimally invasive laparoscopic technique is the best approach for the surgical management of kidney tumours.

## Figures and Tables

**Table 1:** Characteristics of patients undergoing surgical treatment of renal cell carcinoma at Sultan Qaboos University Hospital, Muscat, Oman (N = 56)

Characteristic	n (%)
**Age in years**	56
**Gender**	
Male	34 (60.71)
Female	22 (39.29)
**Nationality**	
Omani	47 (83.63)
Expatriate	9 (16.07)
Smoking	18 (32.14)
**Body mass index status**	
Normal	18 (32.14)
Overweight	22 (39.28)
Obese (BMI >30)	16 (28.58)
**Laterality**	
Left	31 (55.35)
Right	25 (44.65)
**Clinical presentation**	
Incidental findings	31 (55.35)
Gross haematuria	3 (5.35)
Palpable mass	5 (8.92)
Abdominal/flank pain	17 (30.35)
**Comorbidity**	
Diabetes mellitus	21 (37.5)
Hypertension	19 (33.93)
Chronic kidney disease	03 (5.35)
**ASA score**	
1	18 (32.14)
2	22 (39.28)
3	15 (26.78)
4	1 (1.78)

BMI = body mass index; ASA = American Society of Anaesthesiology.

**Table 2:** Comparison of patient characteristics and surgical outcomes of patients undergoing laparoscopic and open procedure for treatment of renal cell carcinoma (N = 56)

Variable	Type pf approach, mean ± SD		*P* value
	Laparoscopic (n = 34)	Open (n = 22)	
Age in years	53.82 ± 13.44	56.23 ± 15.00	0.535
Tumour size in cm	6.25 ± 3.16	9.23 ± 5.20	0.022
Estimated blood loss in mL	351.76 ± 400.98	512.95 ± 616.20	0.405
Surgery time in min	205.00 ± 73.37	217.82 ± 161.87	0.355

SD = standard deviation.

**Table 3:** Tumour, Node, Metastasis staging of patient undergoing laparoscopic and open procedure for treatment of renal cell carcinoma (N = 56).

TNM stage	n (%)
**Primary tumour**	
T1a	15 (26.78
T1b	14 (25.00)
T2a	3 (5.35)
T2b	6 (10.71)
T3a	10 (17.85)
T3b–T3c	6 (10.71)
T4	2 (3.57)
**Regional lymph nodes**	
N0	54 (96.43)
pN1	2 (3.57)
**Distant metastasis**	
M0	54 (96.43)
M1	2 (3.57)

TNM = Tumour, Node, Metastasis; p = pathological stage.
